# Association of subclinical inflammation, glycated hemoglobin and risk for obstructive sleep apnea syndrome

**DOI:** 10.1590/S1679-45082017AO3900

**Published:** 2017

**Authors:** Carolina Vicaria Rodrigues D’Aurea, Bruno Gion de Andrade Cerazi, Antonio Gabriele Laurinavicius, Carolina Castro Porto Silva Janovsky, Raquel Dilguerian de Oliveira Conceição, Raul D Santos, Márcio Sommer Bittencourt

**Affiliations:** 1 Hospital Israelita Albert Einstein, São Paulo, SP, Brazil.; 2 Faculdade Israelita de Ciências da Saúde Albert Einstein, Hospital Israelita Albert Einstein, São Paulo, SP, Brazil.

**Keywords:** Sleep apnea, obstructive, Hemoglobin A, glycosylated, Cardiovascular diseases, Inflammation

## Abstract

**Objective:**

To investigate the inter-relation between high sensitivity C-reactive protein and glycated hemoglobin in prediction of risk of obstructive sleep apnea.

**Methods:**

We included all individuals participating in a check-up program at the Preventive Medicine Center of *Hospital Israelita Albert Einstein* in 2014. The Berlin questionnaire for risk of obstructive sleep apnea was used, and the high sensitivity C-reactive protein and glycated hemoglobin levels were evaluated.

**Results:**

The sample included 7,115 participants (age 43.4±9.6 years, 24.4% women). The Berlin questionnaire showed changes in 434 (6.1%) individuals. This finding was associated with high sensitivity C-reactive protein and glycated hemoglobin levels (p<0.001). However, only the association between the Berlin questionnaire result and glycated hemoglobin remained significant in the adjusted multivariate analysis, for the traditional risk factors and for an additional model, including high-density lipoprotein cholesterol and triglycerides.

**Conclusion:**

The glycated hemoglobin, even below the threshold for diagnosis of diabetes, is independently associated with obstructive sleep apnea syndrome, even after adjustment for obesity and C-reactive protein. These findings suggest a possible pathophysiological link between changes in insulin resistance and obstructive sleep apnea syndrome, independently from obesity or low-grade inflammation.

## RESUMO

**Objetivo:**

Investigar a inter-relação entre proteína C-reativa de alta sensibilidade e hemoglobina glicada na predição do risco de apneia obstrutiva do sono.

**Métodos:**

Foram incluídos todos os indivíduos participantes do programa de *check-up* do Centro de Medicina Preventiva Hospital Israelita Albert Einstein em 2014. Foi aplicado o questionário de Berlin sobre risco de apneia do sono, e avaliadas as dosagens de hemoglobina glicada e proteína C-reativa de alta sensibilidade.

**Resultados:**

Foram incluídos 7.115 participantes (idade 43,4±9,6 anos, 24,4% mulheres). A prevalência de alteração no questionário de Berlin foi de 434 (6,1%). A alteração do questionário de Berlin associou-se positivamente aos resultados da proteína C-reativa de alta sensibilidade e da hemoglobina glicada (p<0,001). No entanto, apenas a associação entre o resultado do questionário de Berlin e a hemoglobina glicada permaneceu significativa na análise multivariada ajustada tanto para fatores de risco tradicionais quanto para um modelo adicional, que incluiu também lipoproteína de alta densidade-colesterol (HDL-c) e triglicérides.

**Conclusão:**

A hemoglobina glicada, mesmo em valores abaixo do critério diagnóstico para *diabetes mellitus*, está associada de forma independente ao risco para síndrome da apneia obstrutiva do sono, mesmo após ajuste para obesidade e proteína C-reativa. Estes achados sugerem possível ligação fisiopatológica entre alterações na resistência insulínica e a síndrome da apneia obstrutiva do sono, que independe da obesidade ou inflamação de baixo grau.

## INTRODUCTION

Obstructive sleep apnea syndrome (OSAS) is a sleep disorder characterized by episodes of partial or total obstruction of airflow in the upper airways, which may lead to changes in arterial oxygen saturation and poor sleep quality. This condition can cause severe physical and cognitive effects, such as excessive drowsiness and impaired memory. However, the most concerning aspect is the association of OSAS, cardiovascular diseases and *diabetes mellitus* (DM).^1,2^

Since OSAS patients show increased inflammatory markers, subclinical inflammation has been suggested as the pathophysiological explanation for this connection. Nonetheless, since patients with *diabetes mellitus* are more likely to have OSAS, subclinical inflammation and cardiovascular diseases,^3-5^ *diabetes mellitus* may possibly be a confounding factor in the association of OSAS and cardiovascular disease.

Although several studies have suggested that the association of *diabetes mellitus* and OSAS can further increase cardiovascular risk,^1,2,6,7^ there is no consistent evidence showing that the association of impaired glucose metabolism and OSAS could occur before the onset of DM. Likewise, it is not clear if low-intensity inflammation has any implication on the risk of developing OSAS in patients without *diabetes mellitus*.

## OBJECTIVE

To investigate the interrelation between high-sensitivity C-reactive protein and glycated hemoglobin in predicting the risk of obstructive sleep apnea.

## METHODS

This is a cross-sectional epidemiological study carried out at the Center for Preventive Medicine of *Hospital Israelita Albert Einstein*. The study was approved by the Ethics Committee under record 1.011.818, CAAE: 42387814.0.0000.0071.

All adult patients visiting the unit for a check-up between January and December 2014 were assessed; laboratory tests were performed for glycated hemoglobin (HbA1c), high-sensitivity C-reactive protein (hsCRP) and lipid profile; patients completed the Berlin questionnaire and were seen by a dietitian and a physical therapist. Patients under 18 and diabetics were excluded. Patients treated with oral antidiabetic agents and subjects with glucose and/or HbA1c levels within diabetic ranges were considered diabetic.

Clinical laboratory tests were performed after 12 hours of fasting. Total cholesterol, high-density lipoprotein cholesterol (HDL-c), triglycerides (TG), glucose (mg/dL) and HbA1c levels were determined by enzyme methods on *in vitro* platforms (Johnson & Johnson Clinical Diagnostics, USA). Low-density lipoprotein cholesterol (LDL-c) was calculated by the Friedewald equation for TG <400mg/dL. Fasting glucose was considered abnormal at levels between 100mg/dL and 126mg/dL. As for HbA1c, values between 5.7% and 6.4% were considered pre-diabetic, and values equal to or greater than 6.5% were considered diabetic, as recommended for clinical practice.^8^ Since diabetic individuals were excluded from the study, 5.7% was the cutoff point for HbA1c. C-reactive protein (CRP) levels in mg/L were determined by immunoturbidimetry (Dade-Boehring, USA). Subclinical inflammation was considered present when CRP >2mg/L, based on previous studies that used this marker to define subclinical atherosclerosis.^9^

Subjects were asked about their history of dyslipidemia (prior diagnosis or use of hypolipidemic drugs), high blood pressure (previous diagnosis of hypertension or use of antihypertensives), diabetes (previous use of antidiabetic agents or fasting glucose >126mg/dL), and previous and current smoking (at least one cigarette in the last 30 days).

The body mass index (BMI) was obtained to classify individuals as eutrophic (BMI <25kg/m^2^), overweight (25 to 29.9kg/m^2^) and obese (≥30kg/m^2^). Waist circumference was measured to assess visceral adiposity. Systolic and diastolic blood pressure was measured according to the American Heart Association criteria,^10^ using a calibrated sphygmomanometer and a suitable cuff size. Blood pressure was measured three times, and a mean value was determined.

The Berlin questionnaire is a validated questionnaire^11^ covering three categories: presence of nighttime snoring (five questions), excessive daytime sleepiness (four questions) and diagnosis of hypertension and/or obesity (one question). Categories are scored as follows: two or more positive answers in categories 1 and 2 and a positive answer to the question in category 3 or BMI >30kg/m^2^. When two or more categories are considered positive, this indicates a high risk for OSAS.

### Statistical analysis

Category variables are described by their absolute and relative frequency. Continuous variables are described as means and standard deviations, except for TG and hsCRP, which are known to have non-normal distribution and are described as medians and quartiles.

Variables were compared using the χ^2^ test, Student’s *t* test and Mann-Whitney test, and multivariate analyzes were conducted using logistic models, and the results are presented as odds ratios (OR) of risk of apnea and 95% confidence intervals. To adjust for possible confounding factors, multivariate models were built. Model 1, adjusted for known cardiovascular risk factors, included age, sex, hypertension, systolic blood pressure and waist circumference. Model 2 also included adjustment for HDL-c and TG. In multivariate models, hsCRP and TG were included after logarithmic transformation to make the variable distribution approximately normal.

Analyzes were performed using the Stata software version 13.0, and the significance level adopted was p=0.01, due to the large number of subjects assessed.

## RESULTS

A total of 7,115 individuals with a mean age of 43.4±9.6 years were enrolled, of which 1,732 (24.4%) were women. The mean HbA1c was 5.46±0.36, and the median hsCRP was 1.2 (interquartile range - IQR: 0.6–2.4). Detailed characteristics of the population are presented in table 1.

**Table 1:** Characteristics of the population according to high-sensibility C-reactive protein

Characteristics	All individuals	hsCRP <2.0	hsCRP ≥2.0	p value
Population	7,115	4,831 (68)	2,284 (32)	
Age	43.4±9.6	43.5±9.5	43.4±9.8	0.69
Female, (%)	1,732 (24.4)	985 (20.4)	747 (32.7)	<0.001
Hypertension, (%)	1,244 (17)	749 (16)	495 (22)	<0.001
Dyslipidemia, (%)	3,411 (48)	2,333 (48)	1,078 (47)	0.39
Smoking				0.01
Current, (%)	1,168 (16)	763 (16)	405 (18)	
Previous, (%)	658 (9)	427 (9)	231 (10)	
HDL-cholesterol	49.9±13.7	50.0±13.3	49.7±14.6	0.28
LDL-cholesterol	117.3±32.8	116.4±32.1	119.1±34.3	0.001
Triglycerides*	111 (81-159)	106 (78-151)	123 (90-172)	<0.001
Waist circumference (cm)	93.4±12.2	91.8±11.0	96.7±13.9	<0.001
BMI	26.5±4.1	25.8±3.5	28.0±4.9	<0.001
Glycated hemoglobin, (%)	5.46±0.36	5.44±0.35	5.49±0.37	<0.001

* Median and quartiles.

BMI: body mass index.

The hsCRP levels higher than 2.0 were associated with the male gender, presence of hypertension, smoking, high BMI and abnormal waist circumference, but not with age or clinical diagnosis of dyslipidemia. Increased hsCRP levels were also associated with high TG and LDL-c, and low HDL-c (Table 1).

Subjects with HbA1c >5.7% were older, predominantly men and had a high prevalence of cardiovascular risk factors (increased waist circumference, high BMI, low HDL-c, and high LDL-c, TG and hsCRP) (Table 2).

**Table 2:** Characteristics of the population as per glycated hemoglobin levels (HbA1c)

Characteristics	HbA1c <5.7%	HbA1c ≥5.7%	p value
Age	41.8±9.2	47.6±9.3	<0.001
Female, (%)	1.331 (26)	401 (20)	<0.001
Hypertension, (%)	699 (14)	545 (27)	<0.001
Dyslipidemia, (%)	2.168 (42)	1.243 (63)	<0.001
Smoking			<0.001
Current, (%)	752 (15)	416 (21)	
Previous, (%)	456 (9)	202 (10)	
HDL-cholesterol	50.6±13.9	48.3±13.2	<0.001
LDL-cholesterol	116.0±32.2	120.4±34.3	<0.001
Triglycerides*	106 (78-150)	127 (92-177)	<0.001
Waist circumference (cm)	91.9±11.7	97.3±12.9	<0.001
BMI	26.0±3.8	27.8±4.6	<0.001
hsCRP	1.3 (0.6-2.6)	1.6 (0.7-3.2)	<0.001

* Median and quartiles.

BMI: Body mass index; hsCRP: high-sensibility C-reactive protein.

**Figure 1:**
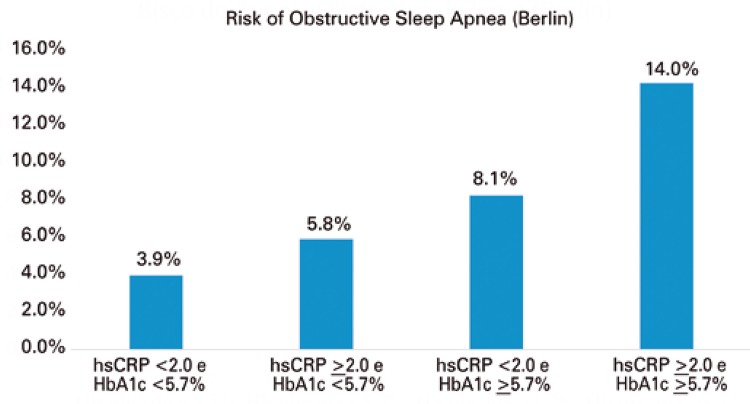
Risk of sleep apnea through the Berlin questionnaire associated to high-sensibility C-reactive protein (hsCRP), glycated hemoglobin (HbA1c) or both

The prevalence of positive results in the Berlin questionnaire was 6.1%. This prevalence increased in direct proportion to increases in hsCRP, HbA1c or both (p<0.001) (Figure 1 ).

While hsCRP and HbA1c, after logarithmic transformation, were associated with abnormal results on the Berlin questionnaire in the univariate analysis, when adjusted for potential confounding factors (age, sex, systolic blood pressure and waist circumference), the association between hsCRP and a high risk for apnea was no longer significant (Table 3).

**Table 3:** Univariate and multivariate analysis of the association of C-high sensitivity reactive protein and glycated hemoglobin and high risk of obstructive sleep risk (Berlin) by logistic regression

	Univariate		Model 1		Model 2	
		
	OR	95%CI	OR	95%CI	OR	95%CI
Ln (hsCRP)	1.38	1.27-1.51	1.08	0.97-1.21	1.07	0.96-1.20
HbA1c	3.92	2.94-5.23	1.67	1.23-2.28	1.61	1.19-2.20

Model 1: age, sex, hypertension, systolic blood pressure, waist circumference; Model 2: model 1 + HDL-cholesterol and triglycerides (after logarithmic transformation); Ln (hsCRP): hsCRP after logarithmic transformation; OR: odds ratio; 95%CI: 95% confidence interval.

On the other hand, HbA1c maintained its association after a similar adjustment (OR: 1.67; 95%CI: 1.23-2.28) and for other components of metabolic syndrome, HDL-c and TG (OR: 1.61; 95%CI: 1.19-2.20).

## DISCUSSION

High hsCRP and HbA1c levels were both associated with a high risk for sleep apnea. However, after adjusting for classic risk factors, such as age, sex, hypertension and waist circumference, only HbA1c showed a positive association with the modified Berlin questionnaire.

Although controversial, the association between obstructive sleep apnea and abnormal HbA1c concentrations has been pointed out in several studies.^12,13^ However, most of them investigated the association between sleep apnea and HbA1c only in diabetic patients. Hermans et al.,^14^ demonstrated that sleep apnea is a frequent comorbidity in diabetic women. Because the present study included only patients without diabetes, we demonstrated that the association between HbA1c and sleep apnea takes place in a continuum: even at values below the diabetic range there is already a direct relationship with a higher risk for obstructive sleep apnea.

However, the mechanism by which HbA1c is associated with OSAS is not clearly defined. Studies in different populations suggest that this association is related to a change in glucose metabolism triggered by hypoxia caused by OSAS, regardless of the presence of diabetes or impaired fasting glucose.^15^ Some studies also suggest that this phenomenon is associated with the severity of OSAS even in non-diabetic individuals.^13,16^ It has also been reported that OSAS can lead to insulin resistance and poor glycemic control, increasing the risk for cardiovascular disease.^17,18^

Despite this possible pathophysiological connection, *diabetes mellitus* and obstructive sleep apnea share some risk factors (such as obesity, for example) and may occur as a cluster. In fact, some data suggests that simultaneous occurrence is associated with increased morbidity and mortality due to a higher incidence of cardiovascular complications.^19^

Mild changes in HbA1c levels are independent predictors for the development of diabetes and long-term cardiovascular mortality also in non-diabetic individuals.^13,20,21^ Since HbA1c testing is recommended in diabetes screening of asymptomatic patients, its result can be useful to assess the risk of sleep apnea. Nevertheless, more studies defining cutoff values or algorithms combining HbA1c results with clinical risk scores for OSAS are needed before these tests can be used in practice to evaluate patients.

There is a consistent relationship between OSAS and systemic inflammation. Hypoxemia and sleep fragmentation caused by OSAS can lead to systemic inflammation documented by changes in hsCRP, interleukin-6 (IL-6) and tumor necrosis factor-alpha (TNF-α) values.^22,23^ Although the association between hsCRP and OSAS was shown in previous studies,^23,24^ the present investigation demonstrated that, after clinical adjustments, the association between hsCRP and increased risk for OSAS was no longer significant. Considering that hsCRP is closely associated with other cardiovascular risk factors,^25^ particularly those associated with metabolic syndrome (low HDL-c, increased TG, increased waist circumference, and high blood pressure), the inclusion of these cofactors in the multivariate model may explain the loss of significance, as previously demonstrated.^26,27^

This study should be interpreted withing the context of its design and its implications in the analysis. First, while most studies investigated the association between the severity of sleep obstructive apnea and HbA1c values, the present investigation assessed the association between the high risk of developing sleep apnea (as assessed by the Berlin questionnaire) and changes in HbA1c and hsCRP concentrations. Also, its cross-sectional design can demonstrate association but not causality, and the findings are only related with a high risk for OSAS but not its actual presence, which would need to be confirmed by polysomnography. Finally, due to the observational nature of the analysis, it is impossible to rule out the existence of confounding factors not included in multivariate models used in the present analysis.

## CONCLUSION

Glycated hemoglobin, even at levels below the diabetic range, is independently associated with increased risk for obstructive sleep apnea syndrome, albeit adjusted for obesity and high-sensitivity C-reactive protein. These findings point to a possible pathophysiological link between impaired insulin resistance and obstructive sleep apnea syndrome, independent of obesity or low-grade inflammation.
